# Interactive Effects of Swimming High-Intensity Interval Training and Resveratrol Supplementation Improve Mitochondrial Protein Levels in the Hippocampus of Aged Rats

**DOI:** 10.1155/2022/8638714

**Published:** 2022-12-10

**Authors:** Maryam Amirazodi, Farhad Daryanoosh, Amin Mehrabi, Abbasali Gaeini, Maryam Koushkie Jahromi, Mohsen Salesi, Amir Hossein Zarifkar

**Affiliations:** ^1^Department of Sport Sciences, Shiraz University, Shiraz, Iran; ^2^Neuroscience Research Center, Institute of Neuropharmacology, Kerman University of Medical Sciences, Kerman, Iran; ^3^Department of Sport Science, Kish International Campus, University of Tehran, Kish, Iran; ^4^Department of Exercise Physiology, Faculty of Physical Education, University of Tehran, Tehran, Iran; ^5^School of Nursing, Larestan University of Medical Sciences, Larestan, Iran

## Abstract

Mitochondrial dysfunction and increased oxidative stress cause damage to cells which can lead to the aging process and age-related diseases. Antioxidants such as resveratrol and high-intensity exercise can benefit oxidative damage prevention. This study is aimed at evaluating the effects of swimming high-intensity interval training and resveratrol on mitochondrial metabolism key proteins, SIRT5, SOD1, and PDH-E1*α*, and the level of NAD^+^ as a cofactor in the deacetylation process in aged rat hippocampus. Forty-five male Wistar rats, aged 20 months, were randomly divided into five groups: control (C), Swimming High-Intensity Interval Training (HIIT) (S-HIIT), Swimming HIIT with resveratrol supplementation (S-HIIT-R), resveratrol supplementation (R), and solvent of resveratrol supplementation (SR). S-HIIT and resveratrol groups performed the exercise and received resveratrol (10 mg/kg/day, gavage) for six weeks. Western blot analysis was performed to determine the protein level in the hippocampus. The amount of SIRT5 and SOD1 proteins in the hippocampus increased. S-HIIT with resveratrol or resveratrol alone increased the PDH-E1*α* level significantly. The amount of NAD^+^ was analyzed by assay kit that was reduced in S-HIIT, S-HIIT-R, and SR groups compared to controls. The results showed that resveratrol and S-HIIT attenuated the age-related brain changes by increasing the expression of SOD1 and SIRT5 and reducing the level of NAD^+^ in the hippocampus. Considering these findings, S-HIIT and resveratrol supplementation could be proposed as strategies to attenuate age-related brain changes. Resveratrol alone and exercise through the regulation of crucial proteins and cofactors can influence mitochondrial metabolism and oxidative stress in the hippocampus of aged rats.

## 1. Introduction

An emerging consensus is that aging is a multifactorial-progressive and dynamic process, which leads to a decline in the functional maintenance of homeostasis in tissues and an increasing tendency to neurodegenerative diseases [1, 2]. Mitochondrial dysfunction was brought to attention in aging biology studies due to the central role of mitochondria in producing endogenous reactive oxygen species through oxidative stress in cells [3, 4]. Aging and mitochondrial dysfunction also are known to particularly, through oxidative damage and disruption of anabolic processes, affect neurons and glial cells in different regions of the brain such as the hippocampus and cortical circuitries [5, 6]. One of the anabolic processes mainly affected by mitochondrial dysfunction is the urea cycle, which is responsible for the detoxification of ammonia during the metabolism of amino acids [7]. In mammals, seven sirtuins (Sirt1–Sirt7) act as sensors and regulators of metabolic status [8]. Sirtuins influence metabolism and anabolic processes initiated by mitochondria in response to physiological processes that lead to changes in energy or ROS levels [9, 10]. It has been contended that downexpression of SIRT5 is associated with molecular brain aging that may be due to increased mitochondrial dysfunction with increasing age [11]. Unlike other deacetylases, the sirtuin family needs NAD^+^ as a cofactor in deacetylation [12].

In addition to a decline in the ability of cells to scavenge ROS, aging is linked to increases in the mitochondria's ability to release superoxide anions [13]. It appears that it can be converted to other forms through a complex antioxidant defense mechanism by superoxide dismutase (SOD) [14]. Gene expression studies in aged rodents have revealed that the antioxidants SOD1 play a vital role in response to oxidative damage during aging [15, 16]. It was highlighted that mice with knockdown of SOD1 have a 30% reduction in lifespan compared to normal mice and accelerated age-related diseases [17–19]. Because oxidative stress induces brain aging, it has been proposed that the use of antioxidants has an influential role in preventing or attenuating age-related changes in the brain [20].

Another essential protein is pyruvate dehydrogenase (PDH), which links glycolysis to tricarboxylic acid (TCA). PDH activity is regulated through reversible phosphorylation in the E1*α* subunit of the PDH enzyme, decreasing pyruvate use in the TCA cycle [21]. It has been revealed that aging leads to phosphorylation of E1*α* (catalytic subunit of E1) PDH enzyme and PDH inhibition which consequently restrains acetyl-CoA production of pyruvate in mitochondria [22, 23].

Previous studies have shown that HIIT can reduce hippocampal oxidative stress by decreasing lipoperoxidation and inflammatory markers and increasing BDNF gene expression [24]. It has also been reported that moderate-intensity exercise reduces oxidative stress and inflammation [25, 26].

Moderate-intensity exercise has also been reported to reduce oxidative stress and inflammation [27, 28]. Resveratrol (3,5,4′-trihydroxystilbene) has recently attracted intense scientific and public interest, mainly due to its different biological properties, including antioxidant, neuroprotective, and anti-inflammation effects [29]. In rodents, resveratrol has been demonstrated to improve skeletal muscle biogenesis, insulin sensitivity, and exercise performance [30–32]. The ability of resveratrol to alter physiological performance has recently attracted interest. Resveratrol has been reported to delay weariness by inhibiting lipid peroxidation, despite contradictions in research on the subject. It has been thought that resveratrol promotes hepatic cell regeneration, regulates glucose metabolism, and protects liver glycogen reserves that are depleted during physical exercise. As a result, the link between resveratrol and exercise is becoming increasingly popular [33].

The hippocampus, a limbic system structure, is affected by aging and mitochondrial dysfunction. It has been also demonstrated that this brain region responds the most to exercise, especially high-intensity interval training (HIIT) [34]. That is why it has received particular attention in metabolisms and aging studies. Despite finding the neuroprotective effect of intensive exercise and antioxidant reagents on attenuating the age-related processes in previous studies, the molecular mechanisms of aging have not been fully understood. Therefore, for further studying the effects of intensive exercise and antioxidant reagents on mechanisms involved in aging, we undertook to observe the effects of swimming high-intensity interval training and resveratrol on the protein levels of SIRT5, SOD1, and PDH-E1*α* and also the level of NAD^+^ in the aged rat hippocampus.

## 2. Materials and Methods

### 2.1. Animals and Grouping

In this developmental study, 45 male 20-month-old Wistar rats (weighing 400-450 g) were purchased from the Neuroscience Research Center of Kerman University of Medical Sciences. Rats were maintained with controlled periods of the 12:12 h light-dark cycle (lights on 07:00–19: 00 h) at the temperature 22-24°C. All rats had free access to both water and food. Tap water and food pellets were provided ad libitum. Rats were randomly divided into five groups (*n* = 9 in each group) and named as control or C group, Swimming High-Intensity Interval Training or S-HIIT group, 1% carboxymethyl cellulose+resveratrol supplementation or SR, S-HIIT+Resveratrol supplementation or S-HIIT-R, and Resveratrol supplementation or R. Twenty-months rats underwent the novel object recognition test to cognitive evaluation (as an aging indication) of rats and also for noninterference of that evaluation with exercise protocol. In addition, the open field test was used to evaluate the lack of motion impairment in 20-months rats. Finally, 45 male 20-months rats (350-450 g) whose novel object recognition test results were negative and had no motion impairment were included in the current study [35].

### 2.2. Exercise Protocol and Resveratrol Treatment

**Figure 1:**
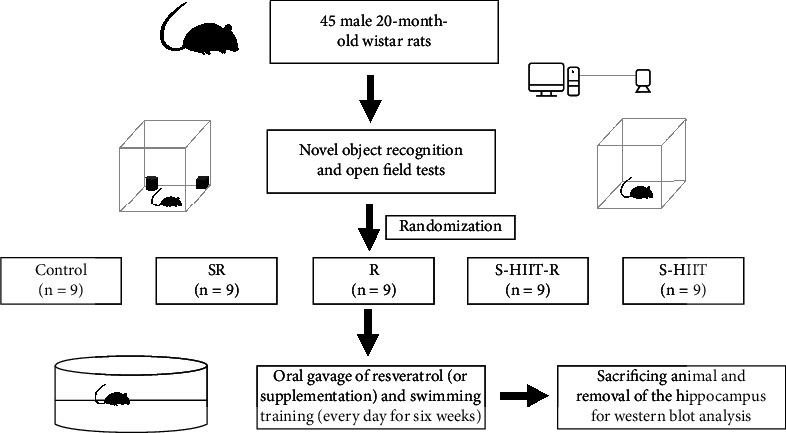
The schematic experimental protocol.

Trained rats were subjected to swimming exercises described by Terada et al. [36]. During the first bout, the rat bore a weight equivalent to 9% of their body weight, and 1% was incrementally increased by 1% of the body weight each week so that rats in the sixth week bearing a weight equivalent to 14% of their body weight [37]. All rats swam in a pool with a 180 cm diameter and 80 cm depth [38]. Water temperature was maintained at room temperature during swimming training. S-HIIT-R rats performed the swimming (from 4:00 PM to 8:00 PM and under the red light to minimize stress responses) and uptake the 1% carboxymethyl cellulose-soluble resveratrol through gavage every day for six weeks. R group rats were gavaged ten cc of supplementation of resveratrol (Serva-10700-USA) solution in 1% oral carboxymethyl cellulose. Aged rats of the C group did not undergo swimming exercises and were untrained. Rats in groups of S-HIIT-R, SR, and R were gavaged from 8:00 AM to 9:00 AM; hence, S-HIIT-R rats were trained at least 8 hours after gavaging to minimize the stress caused by gavaging [39]. To evaluate the intensity of training in the first and third sessions of each week, the blood lactate of the S-HIIT and S-HIIT-R groups was measured after the 14th bout through the circulation blood sample from the tail vein using Lactate Scout (EKF-Germany) [40]. Each rat's swimming speed and traveled distances were determined using an intelligent video tracking system (Noldus Ethovision ® system, Netherland, version 7). Two days after the last training session, to minimize the acute effects of exercise training and predisposing factors for stress, the rats underwent light anesthesia by desiccator attached to a carbon dioxide capsule and the rats were then immediately sacrificed [41]. Afterward, the brains were separated, the hippocampus was isolated from the whole brain, placed on ice, and then fixated using liquid nitrogen (Figure 1).

### 2.3. Western Immunoblotting

As previously described, the hippocampus was collected and prepared for western blotting [42]. Samples were homogenized in RIPA buffer supplemented with inhibitors aprotinin (A1153 sigma), phenylmethylsulfonyl fluoride (P7626 sigma), leupeptin (L2023 sigma), and sodium orthovanadate (S450243 sigma). Samples were incubated for an hour on ice. The lysates were centrifuged (Eppendorf-USA) at 14,000 g for 20 minutes at 4°C. After transferring the supernatant to a new microtube, the appropriate concentration of the tissue protein was checked using the Bradford method. 4% of the sample buffer was added to the supernatant and then the supernatant was heated at 95°C for five minutes to denature the protein. Equal amounts of protein samples were loaded on 12.5% gradient polyacrylamide gel SDS-PAGE and run at 80-100 volts in an electrophoresis buffer. Separated proteins were transferred to polyvinylidene difluoride (PVDF) membranes (0.45 mm sc-3723) over 80 minutes at 220 mA in the transfer buffer. The membranes were washed in tris-buffered saline with tween 20 (TBST) for five minutes and blocked in a 5% blocking solution for two hours. Primary antibodies (SIRT5 (AV32391 sigma), SOD1 (AV45752 sigma), and PDH-E1*α* (ABS204 sigma), produced in rabbit), 1/5000 dilution, were then applied (under incubation) to blots overnight in 4^o^C, and then washed (using TBST) three times and 10 minutes each time, and secondary antibodies (horseradish peroxidase-conjugated anti-rabbit antibody), 1/100,000 dilution, applied for 1-2 hours at 24°C. Membranes were again washed with TBST and incubated to ECL (GERPN2232). The light emitted from the luminescence reaction was recorded on BioMax film (Roche-000000011666657001) in the darkroom. After that, using photo-processing solutions, the images appeared. Film densitometry was determined using Image J software.

### 2.4. NAD^**+**^ Measurement

NAD^+^ level in hippocampus tissue was determined using the colorimetric method by NAD^+^/NADH assay kit described by the manufacturer (Abcam, Cat #65348, UK) [43]. Hippocampal tissues were homogenized in ice-cold and centrifuged for 5 minutes at 4°C at 16000 g. The supernatant was filtered via a microspin column with a 10 kDa cutoff to separate the NADH-consuming enzymes. In a 96-well microplate, the heated (NADH) and unheated (total NAD(H) samples were mixed separately for five minutes using NAD^+^/NADH cycling assay mix. After two hours, the color was developed with NADH developer solution, and the absorbance was measured at 450 nm using a microplate reader. An aliquot of homogenized material was utilized to assess the concentration before ultrafiltration. Before ultrafiltration, an aliquot of homogenized material was used to test the protein concentration using the conventional bio-rad procedure of measuring the absorbency at 595 nm. Based on conventional NADH measurements, the amount of NAD^+^ was represented in pmol per mg protein.

### 2.5. Statistical Analysis

All statistical analyses were performed using SPSS (version 20; Chicago, IL). The normality of the distribution of research variables was examined using Shapiro–Wilk test. One-way and two-way analyses of variance (ANOVAs) were used to analyze the data. When a statistically significance difference was found, Tukey's post hoc multiple comparison test was used to determine points of significant difference. All values in the present study were expressed as mean ± SEM, and a *P* value less than 0.05 was defined as significant.

## 3. Results

The body weight of the rats in all groups in the 1st, 4th, and 6th weeks was shown in Table 1, respectively. Moreover, repeated measures of the present study indicated the average changes in speed and distance in S-HIIT and S-HIIT-R groups in each bout and section of aged rat (M ± SE) in the 1st, 4th, and 6th weeks reported in Table 2. Moreover, the average changes of lactate amount in control, S-HIIT, and S-HIIT-R groups in the 3rd and 6th weeks are reported in Table 3.

**Figure 2:**
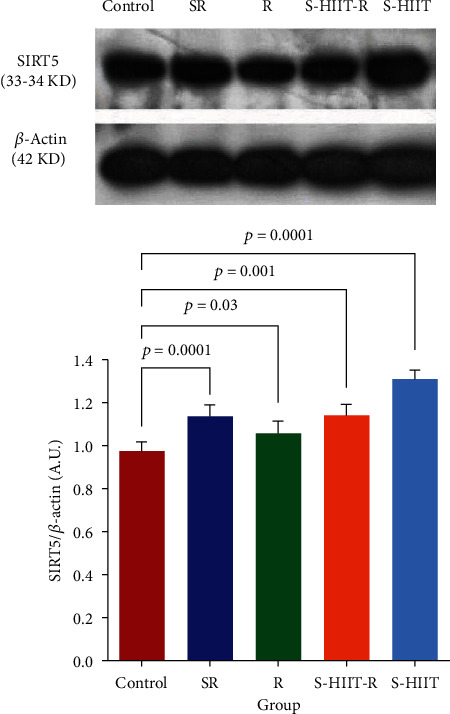
Comparison of the SIRT5 level in the study groups. (a) Immunoblotting images of SIRT5 protein and *β*-actin protein are shown as control loading in the hippocampus tissue. (b) The SIRT5 protein quantified bands are against the control loadings. The values are shown as mean ± SEM. SIRT5 protein level in the SR group (*P* = 0.0001), R group (*P* = 0.003), S-HIIT-R group (*P* = 0.001), and S-HIIT group (*n* = 0.0001) compared to the control group has increased; (*n* = 9 per group). Control (C), swimming HIIT (S-HIIT), swimming HIIT with resveratrol supplementation (S-HIIT-R), resveratrol supplementation (R), and solvent of resveratrol (SR). ^∗^Because the images were taken with the device, the original images of blots are not full-length.

**Figure 3:**
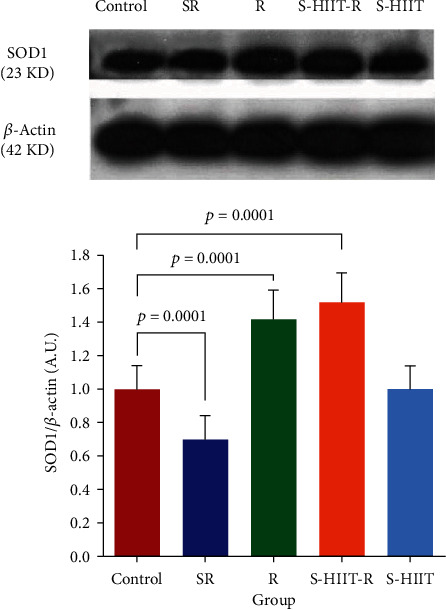
Comparison of the SOD1 level in the study groups. (a) Immunoblotting images of SOD1 protein and *β*-actin protein are shown as control loading in the hippocampus tissue. (b) The SOD1 protein quantified bands are against the control loadings. The values are shown as mean ± SEM. The SOD1 protein level in R and S-HIIT-R groups (*P* = 0.0001) compared to the control group has increased and in the SR group (*P* = 0.0001) has decreased compared to the control group; (*n* = 9 per group). Control (C), swimming HIIT (S-HIIT), swimming HIIT with resveratrol supplementation (S-HIIT-R), resveratrol supplementation (R), and solvent of resveratrol (SR). ^∗^Because the images were taken with the device, the original images of blots are not full-length.

**Figure 4:**
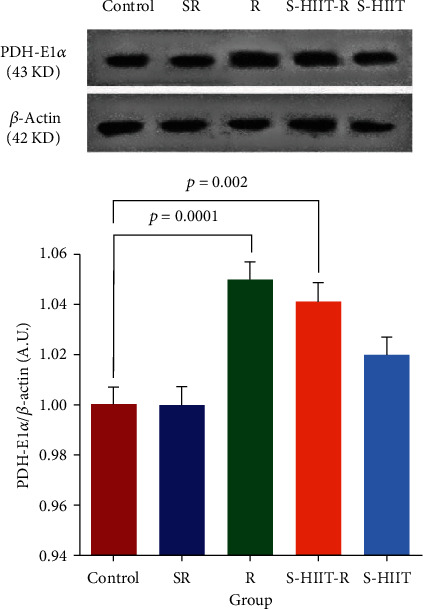
Comparison of the PDH-E1*α* level in the study groups. (a) Immunoblotting images of PDH-E1*α* protein and *β*-actin protein are shown as control loading in the hippocampus tissue. (b) The PDH-E1*α* protein quantified bands are against the control loadings. The PDH-E1*α* protein level in R (*P* = 0.007) and S-HIIT-R (*P* = 0.047) groups, compared to the SR group has increased. Swimming S-HIIT has increased PDH-E1*α* protein level compared to the control group but not significantly (*P* = 0.383); (*n* = 9 per group). Control (C), swimming HIIT (S-HIIT), swimming HIIT with resveratrol supplementation (S-HIIT-R), resveratrol supplementation (R), and solvent of resveratrol (SR). ^∗^Because the images were taken with the device, the original images of blots are not full-length.

After confirming the normality of the distribution of research variables with the Shapiro–Wilk test, it has been shown that the SIRT5 protein level of aged rats' hippocampus in S-HIIT, S-HIIT-R, R, and SR groups compared to the control group significantly increased (*P* < 0.05). In addition, the SOD1 protein level increased significantly in S-HIIT-R and R groups and significantly decreased in the SR group (*P* < 0.05) (Figure 2). In response to whether the studied protein levels statistically are significant between groups, a one-way analysis of variance of the current study revealed a statistically significant difference for SIRT5 protein (*P* = 0.0001) and SOD1 protein (*P* = 0.0001) between all groups. Tukey's post hoc test results have also shown a statistically significant difference for SIRT5 between SR and S-HIIT groups (*P* = 0.0001), between R and S-HIIT groups (*P* = 0.0001), and S-HIIT and S-HIIT-R groups. In addition, there is a statistically significant difference for SOD1 between all intervention groups (*P* < 0.05) (Figure 3). The results have shown a significant difference for SIRT5 and SOD1 between SR and control groups (*P* = 0.0001). One-way analysis of variance of the present study indicated that average amounts of E1(*α*) catalytic subunit of pyruvate dehydrogenase enzyme and Tukey's post hoc test in R and S-HIIT-R groups increased significantly compared to SR group (*P* = 0.007, *P* = 0.047, and *F* = 5.476, respectively) (Figure 4).

**Figure 5:**
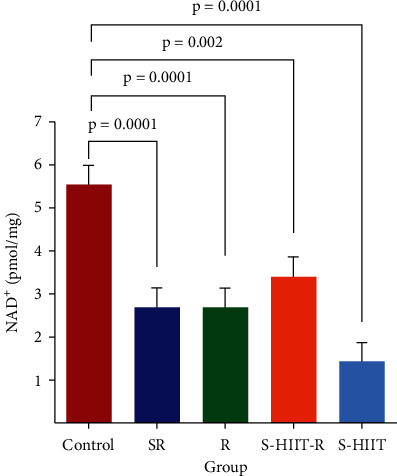
Comparison of the NAD^+^ level in the study groups. The values are shown as mean ± SEM. The NAD^+^ level in SR, R, and S-HIIT groups (*n* = 0.0001) and in the S-HIIT-R group (*P* = 0.002) compared to the control group has decreased. The values are shown as mean ± SEM; (*n* = 9 per group). C: control; S-HIIT: swimming HIIT; S-HIIT-R: swimming HIIT with resveratrol; R: resveratrol supplementation; SR: solvent of resveratrol.

It was also found that the amount of PDH-E1*α* in the SR group, compared to the control group did not have a significant difference (*P* = 0.99). Moreover, it was also realized that the amount of this subunit in the swimming HIIT group compared to the control group increased but not significantly (*P* = 0.383). One-way analysis of variance of the current study revealed a statistically significant difference in NAD^+^ level (*P* = 0.005) between all groups (Figure 5). It has been shown that the NAD^+^ level of aged rats' hippocampus significantly reduced in all groups compared to the control (*P* < 0.05). Tukey's post hoc test showed a statistically significant difference for NAD^+^ between S-HIIT and S-HIIT-R groups (*P* = 0.003).

**Figure 6:**
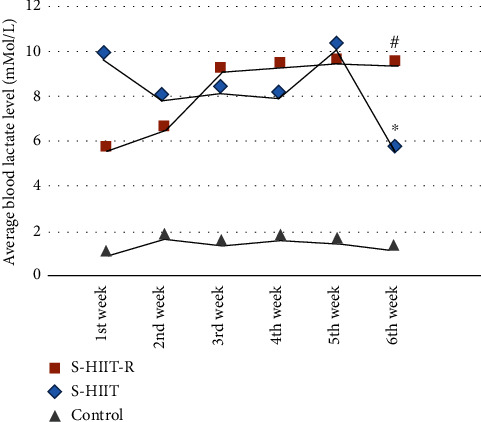
Comparison of the average blood lactate level between S-HIIT and S-HIIT-R groups with the C group. The amount of blood lactate in the 6th week in the aged rats of the S-HIIT group declined compared to the first week ^∗^(*P* = 0.0001). In contrast, the amount of blood lactate in the 3rd and 6th weeks in the S-HIIT-R group aged rats increased ^#^(*P* = 0.0001); (*n* = 9 per group). C: control; S-HIIT: swimming HIIT; S-HIIT-R: swimming HIIT with resveratrol.

Additionally, our results have shown that the amount of average blood lactate level (mMol/L) in the 6th week in the aged rats of the S-HIIT group declined in comparison with the 1st and 3rd weeks (*P* = 0.0001) (Figure 6). In contrast, the average blood lactate level in the S-HIIT-R group aged rats in the 3rd and 6th weeks increased (*P* = 0.0001).

## 4. Discussion

This study examined the effects of swimming high-intensity interval training and resveratrol on the protein levels of SIRT5, SOD1, and PDH-E1*α* in the aged rat hippocampus. The present study showed that resveratrol alone or with swimming high-intensity interval training could increase the SIRT5, SOD1, and PDH-E1*α* proteins of aged rats' hippocampus. In addition, we found that NAD^+^ level was reduced in all groups. Also, our results showed that resveratrol with swimming high-intensity interval training increases blood lactate within six weeks. There are differences between the control and solvent of resveratrol groups in the protein levels of SIRT5 and SOD1, which could be due to the gavage-induced stress.

This study demonstrated, for the first time, that swimming high-intensity interval training and resveratrol increases the protein levels of SOD1 and SIRT5 in the hippocampus region. Increasing SOD1 leads to decreasing oxidative damage. In addition, increasing SIRT5 through deacetylation increases the enzymatic activity of carbamoyl phosphate synthetase 1 (CPS1). The amount of PDH-E1*α* in the S-HIIT-R and resveratrol supplementation groups increased significantly. It was also indicated that in the S-HIIT group, the amount of PDH-E1*α* increased, but it was not significant. Considering these findings, swimming high-intensity interval exercise training and resveratrol may have been proposed as strategies to attenuate age-related brain changes. Evidence is now rapidly emerging showing brain aging of the mammalian is characterized by increased biomarkers of oxidative stress and accumulation of damage to DNA or protein, ultimately leading to neuronal dysfunction [44]. It has been shown that resveratrol supplementation for eight weeks and 20 mg/kg/day in 20-month-old male rats may lead to morphological changes such as increasing dendritic column density or dendritic length in the hippocampus neurons [45].

Furthermore, it was reported that resveratrol supplementation, by decreasing mitochondrial dysfunction and oxidative damage, maintains its neuroprotective role [46]. These studies support the results obtained in the current study. Besides, In line with these findings, some previous studies have also shown that resveratrol alone and with exercise in aged rats can affect the essential proteins involved in brain mitochondrial metabolism, such as SIRT3 in frontal lobe cell metabolism [47].

Resveratrol affects a variety of proteins and signaling pathways [48]. The current study found that this supplementation increases SIRT5 protein levels in the hippocampus of aged rats, supported by another study performed by Gertz et al. [49]. SIRT5-mediated deacetylation of proteins has been a vital regulator for several cellular processes, including control over energy homeostasis and antiaging action [50]. Another report indicated that the SIRT5, increased by increasing NAD^+^ level, was distributed in the mitochondria, cytoplasm, and nucleus in cerebellar granule cells [51]. In an in vivo and in vitro study conducted by Geng et al., it was highlighted that the SIRT5 protein level is increased in the calorie restriction neurons [52]. Besides, increasing evidence suggests that calorie restriction is an effective strategy to prevent aging in mammalian neurons [53], suggesting that SIRT5 may be a novel antiaging regulation factor that decreases neural defects. In addition, it was reported that the activity of NAD^+^ by increasing the SIRT5 protein level contributes to a broad range of age-related pathophysiologies [54].

Consistent with our results, it has been revealed that transgenic expression of SOD1 causes attenuation of the accumulation of the oxidative damage of DNA and protein in the neurons in aged mice [55] and prevents neurodegeneration [56]. However, there are still unknown mechanisms by which the SOD1 level can increase through resveratrol.

Regarding the results of this study in which the resveratrol supplementation increased the amount of PDH-E1*α* protein in aged rats' hippocampus, and also due to this fact that aging leads to phosphorylation of E1*α* subunit of PDH enzyme and consequently to inhibition of PDH complex [22]. This, as a result, leads to dephosphorylating of PDH-E1*α* and finally increases the activity of PDH enzyme in mitochondria of rat's hippocampus cells. It was also found that high- and low-intensity combined exercise for eight weeks on an ergometer cycle among 60- to 72-year-old men has increased the amount of PDH-E1*α* protein in muscular cells [57]. What is noticeable in both this study and Bienso et al. is that older people can also undergo high-intensity training programs. In untrained people, when VO_2_ reaches a maximum of 60%-70%, and in trained people, the VO_2_ reaches 75%-80%, the lactate threshold is too much. Therefore, the supralactate threshold, special for high-intensity training, has to be noticed in older people. To this end, many studies have shown that aerobic exercise of 60% to 80% VO_2_ max is possible and practical [58]. Also, lactate, a by-product of muscle exercise, enters the brain and is used as an energy source. Moreover, swimming elevates the lactate level in the body and can compensate for the lack or decline of metabolic flexibility caused by the aging process [59].

Inconsistent with these results, recently, it was demonstrated that HIIT decreased the neural survival and memory functions in the rat hippocampus through the downregulation of dynein and KIF5B, respectively [60]. One reason for these conflicting results is that all types of exercise intensity may not cause the same adaptation. Some of the exercise intensity may have detrimental effects on neurons. Intensive exercise may positively and negatively affect the proliferation rate of cells and neurotrophic factor levels in a rat's hippocampus [61]. The mechanism underlying the effect of HIIT on gene expression is not yet fully understood and requires extensive studies in this regard.

## 5. Conclusions

In general, the current study describes some novel effects of resveratrol and S-HIIT on attenuating age-related brain changes by increasing the expression of SOD1 and SIRT5 and decreasing the level of NAD^+^ in the hippocampus. Furthermore, resveratrol and S-HIIT as bioenergetic interventions can affect mitochondrial metabolism through PDH-E1*α* in the hippocampus of old rats. Also, lactate, a by-product of muscle exercise, enters the brain and is used as an energy source. Based on our findings and previous studies, it can be proposed that resveratrol and S-HIIT might improve hippocampal function by influencing these factors.

### 5.1. Study Limitations

In the current study, the limitations must be acknowledged. First, uncontrollable stress (stress during S-HIIT, stress during resting between swimming bouts, stress caused by adding weights to rats' tails, stress caused by gavaging, and stress during anesthesia induction) may be confounding factors. It may create a bias in the data obtained. Second, age-related changes also can be one obvious limitation of such studies. A third limitation is the lack of behavioral evaluations due to the possible confounding role of stress resulting from testing. Also, the animals were not sacrificed until 48 hours had passed since the HIIT. This process would affect the production of factors we measured. Finally, this study was conducted on animals; however, HIIT should be undertaken on the aged population who do not have orthopedic problems in human studies.

## Figures and Tables

**Table 1:** The variation of weight (g) in different groups in 1st, 4th, and 6th weeks (M ± SD).

Weight			
Group	First week	Fourth week	Sixth week
C	415.66 ± 34.88	430.77 ± 33.09	425.55 ± 35.21
SR	400.22 ± 31.95	416.56 ± 24.95	427.89 ± 22.33
R	400.22 ± 30.19	392.11 ± 33.57	390.67 ± 34.67
S-HIIT-R	401.44 ± 31.59	388.23 ± 22.11	380.33 ± 17.03
S-HIIT	404.44 ± 30.68	404.67 ± 27.73	405.22 ± 29.95

Note: C: control; S-HIIT: swimming HIIT; S-HIIT-R: swimming HIIT with resveratrol; R: resveratrol supplementation; SR: solvent of resveratrol.

**Table 2:** Average speed and distance in S-HIIT of aged rats in 1st, 4th, and 6th weeks (M ± SD).

	Group	Swimming speed	Swimming distance in each bout	Total swimming distance in each section
		Cm/sec	Cm	Cm
First week	S-HIIT-R	9.70 ± 8	174.54 ± 147	2443.56
	S-HIIT	8.83 ± 9	160.73 ± 163	2250.22

Fourth week	S-HIIT-R	18.75 ± 5	311.66 ± 139	4363.24
	S-HIIT	18.03 ± 5	315.52 ± 125	4347.28

Sixth week	S-HIIT-R	9.95 ± 1	175.96 ± 25	2463.44
	S-HIIT	9.54 ± 1	174.1 ± 71	2443.56

Note: C: control; S-HIIT: swimming HIIT; S-HIIT-R: swimming HIIT with resveratrol.

**Table 3:** Average blood lactate level (mMol/L) of aged rats in S-HIIT in 1st, 3rd, and 6th weeks (M ± SD).

Variable	Group	First week	Third week	Sixth week
Blood lactate level (mMol/L)	C	2.03 ± 0.15	2.00 ± 0.17	2.01 ± 0.12
	S-HIIT-R	8.50 ± 1.79	9.80 ± 0.27^∗^	10.00 ± 0.28^∗^
	S-HIIT	8.68 ± 1.48	8.80 ± 0.51	6.11 ± 0.29^∗^

NOTE: The ^∗^ indicates a significant difference relative to the rest values (*P* < 0.05). C: control; S-HIIT: swimming HIIT; S-HIIT-R, Swimming HIIT with resveratrol.

## Data Availability

The datasets generated and/or analyzed during the current study are available in the G-Node repository (https://gin.g-node.org/Zarifkar/Raw-data.git).

## Abbreviations

ANOVA: One-way analysis of variance
CPS1: Carbamoyl phosphate synthetase 1
HIIT: High-intensity interval training
MOHME: Ministry of Health and Medical Education
NAD^+^: Nicotinamide adenine dinucleotide
PDC: Pyruvate dehydrogenase complex
PDH: Pyruvate dehydrogenase
PDP: Pyruvate dehydrogenase phosphatase
ROS: Reactive oxygen species
SIRT: Sirtuin
SOD: Superoxide dismutase
SPSS: Statistical package for the social sciences
TCA: Tricarboxylic acid cycle.

## Supplementary Materials

Supplementary Materials Supplementary files include graphical abstract of the study.
