# Supplementary figures and images for: Interactive Effects of Swimming High-Intensity Interval Training and Resveratrol Supplementation Improve Mitochondrial Protein Levels in the Hippocampus of Aged Rats
Source: Biomed Res Int. 2022 Dec 10;2022:8638714. doi: 10.1155/2022/8638714 (PMC9759392; doi:10.1155/2022/8638714)

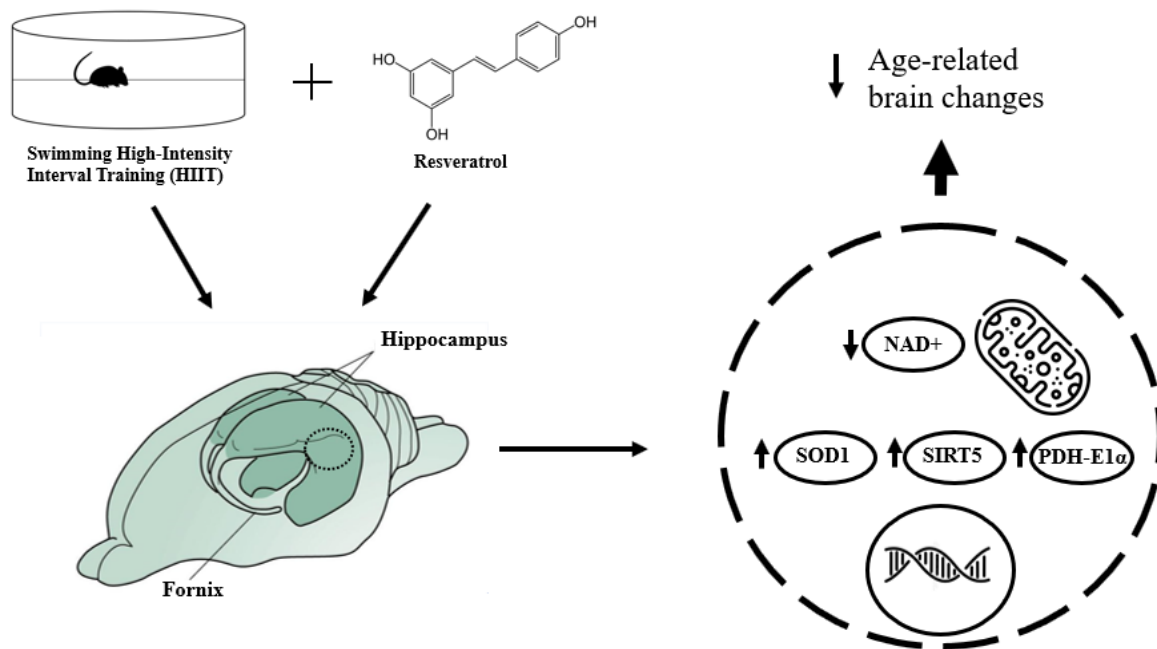

Supplement: Supplementary Materials — Supplementary files include graphical abstract of the study. [file 8638714.f1.pdf]
